# Checkpoint Inhibitors in Multiple Myeloma: Intriguing Potential and Unfulfilled Promises

**DOI:** 10.3390/cancers14010113

**Published:** 2021-12-27

**Authors:** Omar Alkharabsheh, Zachary Trisel, Sunil Badami, Mohammed A. Aljama, M. Hasib Sidiqi

**Affiliations:** 1 Mitchell Cancer Institute, University of South Alabama, Mobile, AL 36604, USA; ztrisel@health.southalabama.edu (Z.T.); sbadami@health.southalabama.edu (S.B.); 2 Department of Oncology, Division of Malignant Hematology, McMaster University, Hamilton, ON L8V 5C2, Canada; aljama@hhsc.ca; 3 Department of Haematology, Fiona Stanley Hospital, Murdoch 6150, Australia; hasib.sidiqi@health.wa.gov.au; 4 Curtin Medical School, Curtin University, Perth 6845, Australia

**Keywords:** multiple myeloma, checkpoint inhibitors

## Abstract

**Simple Summary:**

Multiple myeloma (MM) is a hematological malignancy caused by aberrations in the plasma cell development, resulting in uncontrolled production of plasma cells and or monoclonal proteins. Currently, several treatments are centered around immune-based therapies including immunomodulatory agents, monoclonal antibodies, cellular therapies, and stem cell transplantation. We review the biologic rationale, efficacy, side effects, and limitations of immune-checkpoint inhibitors (ICIs), which have been successful in several solid organ malignancies such as melanoma, kidney, and lung cancers. Recent clinical trials evaluating ICIs as monotherapy and in combination with approved pharmaceutical drugs in MM, have thus far remained underwhelming due to excessive side effects and lack of efficacy in aggressive and advanced cases. There may be a role for ICIs in early forms of MM and preventing recurrence once achieving remission. Side effect mitigation and improved efficacy to combat resistance are needed prior to profoundly impacting the current MM landscape. Clinical studies to answer these questions are ongoing, and we eagerly await this data to determine if ICIs will become a permanent fixture in MM.

**Abstract:**

Immune dysregulation and alteration of the bone marrow microenvironment allowing plasma cells to escape immune surveillance are well-known factors associated with the proliferation of clonal plasma cells and development of multiple myeloma (MM). Whilst immunotherapeutic approaches are now commonplace in a wide spectrum of malignancies, this aberration of myeloma development gives rise to the biological rationale for the use of immune checkpoint inhibitors (ICIs) in MM. However, the initial experience with these agents has been challenging with limited single agent efficacy, significant toxicity, and side effects. Herein, we review the biological and immunological aspects of MM and ICIs. We discuss the basic biology of immune checkpoint inhibitors, mechanisms of resistance, and drug failure patterns, review the published clinical trial data for ICIs in MM, and look towards the future of ICIs for MM treatment.

## 1. Introduction

The current therapeutic landscape for multiple myeloma (MM) is rapidly evolving, with data emerging from numerous clinical trials utilizing various novel therapy combinations. Currently, several clinical trials are in progress and/or reporting results assessing each class of therapy in triplet and quadruplet combinations. In recent years, novel classes of drugs have been identified to treat myeloma, and amongst these immunotherapeutic approaches there has been a standout, with the hematology community eager to see longer-term results for clinical trials utilizing chimeric antigen T-cell therapy (CAR-T). This modality could potentially revolutionize the treatment landscape, making traditional MM therapies obsolete, including autologous stem cell transplantation. Despite these advances in treatment, including cellular therapies, MM remains an incurable malignancy.

The trend over the last decade has been to focus on doublet, triplet, and quadruplet combinations of novel agents with clinical trials aiming to advance the combinations to obtain the Food and Drug Administration (FDA) approval. Nonetheless, one of the most important questions in MM therapy remains unanswered: how to sequence the treatment options in relapsed disease? The National Cancer Comprehensive Network (NCCN) provides numerous combination options with no clear direction for frontline and subsequent treatment [1]. Historically, stage IV melanoma and lung cancers had very poor outcomes, secondary to limited therapeutic options aside from chemotherapy, prior to the advent of immune-checkpoint inhibitors (ICIs). Currently, we have three classes of ICIs approved in clinical practice: cytotoxic T-lymphocyte-associated protein 4 (CTLA-4), programmed cell death protein 1 (PD-1), and programmed death-ligand 1 (PD-L1). They show efficacy, survival advantage, and tolerability in solid malignancies across all age groups [2]. These promising results of ICIs in solid malignancies motivated investigators to study their activity in hematologic malignancies. Whilst successful in Hodgkin’s lymphoma, efficacy in other hematological malignancies such as leukemia and multiple myeloma have been poor [3]. In contrast, other immunotherapeutic approaches to MM have been very successful, including monoclonal antibodies targeting CD38, signaling lymphocytic activation molecule family member 7 (SLAMF7), antibody–drug conjugate for B-cell maturation antigen (BCMA) and CAR-T [4].

Clonal plasma cell proliferation in the marrow is strongly associated with the bone marrow microenvironment and dysregulated immune system. This provides an avenue to treatment failure and drug resistance. However, this is not applicable to extramedullary disease or secondary plasma cell leukemia [5]. Additionally, epigenetic modifiers such as histone deacetylase inhibitors are approved for relapsed myeloma but with a marginal response [6]. Accordingly, it is very difficult to forecast an immunological pathway for treatment response or resistance in a dysfunctional immune system from plasma cell neoplasms.

In this review, we will focus on the basic biology of immune-checkpoint inhibitors, mechanisms of action, and drug failure and resistance patterns, as well as correlate them to the biological and immunological aspects of plasma cell neoplasm. Additionally, we will review the current and published clinical trials evaluating ICIs as mono or combination therapy with other anti-myeloma agents.

## 2. Plasma Cells and Immune Checkpoints

Plasma cells are terminally differentiated B-cells, originating from the post-germinal center. They contribute to the adaptive and humoral immune system by manufacturing different types of antibodies according to antigenic stimuli [7]. Oncogenesis of plasma cells is a complex and multistep process. Whether the initial process starts with the myeloma stem cell, plasmablasts, or in mature plasma cells remains a matter of debate [8].

As in many malignancies, clonal cells go through a dormancy stage, and this is applicable to plasma cell neoplasms as they live longer, compared to plasmablasts, and exhibit a wide spectrum of phenotypes, such as tumor burden in the marrow, the amount of monoclonal protein production (monoclonal gammopathy of undetermined significance (MGUS) vs. smoldering myeloma), and the presence of end organ damage (as in MM), irrespective of the amount of monoclonal proteins. MM can manifest as oligo-secretory disease, non-secretory, or non-producer [9].

The initial genetic trigger for MM primarily involves immunoglobulin heavy chain (IGH) rearrangement and/or hyperdiploidy [10]. Several theories have been proposed to explain the genetic component of progression. However, the fact that MGUS and MM have a genetic similarity raises the question of immune escape phenomena in plasma cell neoplasms [11].

With any discussion regarding immune escape, we need to address the role of the bone marrow microenvironment. It is a complex interaction between clonal plasma cells, the extracellular matrix, effector T-cells, myeloid-derived suppressor cells, and marrow cytokines [12]. Here, we will concentrate on ICIs in plasma cells and their correlation with antigen-presenting cells along with immune escape mechanisms. PD-1 is expressed on T-cells and PD-L1 on antigen-presenting cells including tumor cells, such as the clonal plasma cells in our topic. The ligation of PD-1 and PD-L1 leads to inhibition of T-cell activation against tumor cells [13].

Plasma cells are the major component of humoral immunity because of their ability to produce immunoglobulin to deliver an appropriate response to pathogens or cancer cells upon first exposure and subsequent events. This process works in close collaboration with effector T-cells (T-helper, T-cytotoxic, and T-regulatory) [14]. Immune-checkpoint modulators can stimulate or inhibit the immune system, particularly the adaptive (acquired) immune system, to maintain a self-tolerance state and mitigate an overactive immune system or immune escape. This mechanism is also used by cancer cells to enhance tumor growth, chemotherapy resistance, and self-renewal [15]. B-cells, macrophages, and dendritic cells represent the antigen-presenting cells (APC) that interact with T-cells through the major histocompatibility complex and T-cell receptor (MHC-TCR). Several costimulatory and inhibitory signals, mainly the B7-CD28 family, contribute to the activity of MHC-TCR [16]. Immune inhibition through this pathway leads to clonal plasma cells escaping T-cell immunosurveillance. Preclinical models have shown an activated PD-1 positive T-cell can be used to eradicate myeloma cells as a concept of adoptive T-cell therapy [17]. Another preclinical aspect is to study approaches to overcome T-cell exhaustion through PD-1/PD-L1 pathway inhibition, which was successful to induce response in a myeloma mouse model [18].

## 3. Can Failure of the Immune Checkpoint Explain the Progression of Myeloma?

We have a good understanding of the incidence of progression from monoclonal gammopathy to symptomatic disease from a 40-year long-term follow-up database [19] but not the biology and the triggering points for oncogenic progression, whether clonal evolution or immune evasion or both. Nevertheless, immune evasion contributes to a considerable part of the progression avenues of MGUS to MM [11]. One of these escape mechanisms is the upregulation of inhibitory ligands to immune checkpoints leading to loss of effector T-cell functions [20]. T-cell regulators (T-regs) control the overactive immune system disease and prevent autoimmunity. In MM, it was observed that T-regs with high PD-1 expression are associated with treatment failure. Furthermore, MGUS and MM patients, post-autologous stem cell transplantation, have lower PD-1 expression on T-regs [21].

The SWOG 0120 is a prospective study examining immunological patterns in MGUS and predictors of clinical progression. Of interest to our topic, the study team showed PD-L1 expression is higher on clonal plasma cells and infiltrating T-cells in patients who exhibit clinical progression to symptomatic myeloma [22]. Other reports demonstrated variable expression of PD-L1 on clonal plasma cells at different stages of disease from newly diagnosed MGUS and MM, to patients with persistent measurable residual disease (MRD) post-intensive therapy to relapsed MM, indicating upregulation of PD-L1 is associated with worse outcomes of MM [23]. A greater attention to MRD-positive disease, whether using PD-L1 maintenance can reduce progression, needs further investigation.

## 4. Patterns of Immune Resistance in MM

The ligation of PD-1 and PD-L1 leads to the activation of Src homology 2 domain-containing 1 and 2 (SHP-1/SHP-2) and immunoreceptor Tyrosine-based switch motif (ITSM). These events generate a dephosphorylation process in several kinases, including PI3K-AKT and RAS-MEK-ERK pathways, leading to T-cell inhibition [13]. According to early clinical trials of checkpoint inhibitors used in solid malignancies for patterns of resistance, failure of PD-1/PD-L1 blockade can be secondary to several processes. First, inadequate recognition by CD8+ cells of tumor cells can be from low tumor immunogenicity (less de-differentiation or lower neoantigen presentation) and/or T-cell exclusion from the tumor microenvironment via activation of the B-catenin-Wnt signaling pathway. Second, mutations can occur in the downstream PD-1/PD-L1 blockade pathway, such as mitogen-activated protein kinase (MAPK), phosphatase and tensin homolog (PETN), and phosphatidylinositol 3-kinases (PI3K) rearrangements. Third, T-cells are resistant to interferon-γ. These can lead to either primary treatment failure or acquired resistance [24]. Most MM regimens use a combination of three or more drugs, making it difficult to delineate drug–mechanism resistance. Very few agents are used as monotherapy outside of early phase clinical trials, which limits our understanding of the resistance pathways in MM to either small clinical trials or bench-side experiments. A pilot study for using pembrolizumab in patients with high-risk smoldering myeloma conducted at the MD Anderson Cancer Center, showed stable disease in 85% (*n* = 11) of the patients at 27 months follow-up, and identified a case of a stringent CR. Further molecular characterization of that case revealed a high-risk gene expression profile (GEP-70), 17p. deletion by FISH, and cyclin kinase subunit 1B (CSKS1B) mutation. Additionally, non-responders had T-cell exhaustion, and the responder had interferon gamma genes upregulation, as seen in the melanoma and ICI literature [25].

## 5. Clinical Trial Outcomes Using Immune-Checkpoint Inhibitors in Multiple Myeloma

**Figure 1:**
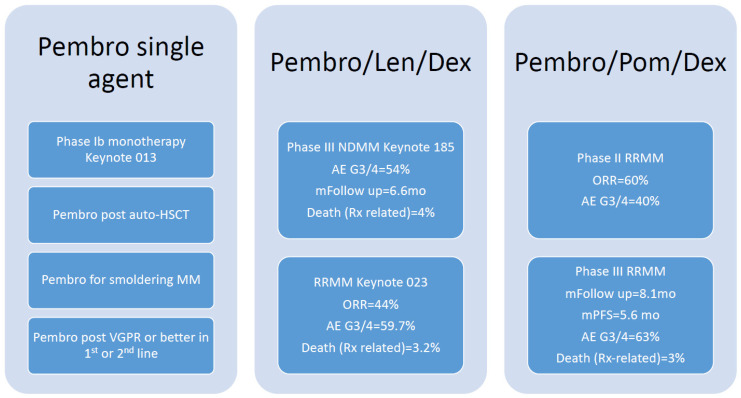
Summary of clinical trials for pembrolizumab in multiple myeloma.

**Figure 2:**
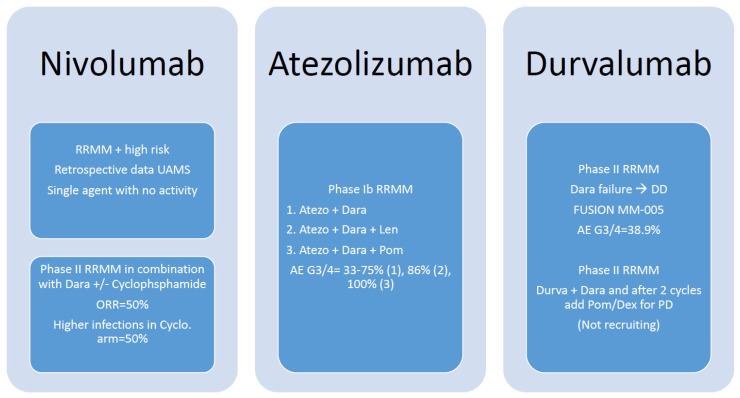
Summary of clinical trials for nivolumab, atezolizumab, and durvalumab in multiple myeloma.

Several early and advanced phase investigational trials have evaluated ICIs, including PD-1- and PD-L1-targeted immuno-oncology agents in various stages of multiple myeloma. A brief schematic illustration of different combination of ICIs in Figure 1 and Figure 2. Keynote 013 is an early phase Ib, evaluated pembrolizumab monotherapy in patients with advanced hematologic malignancies. Thirty enrolled participants with relapsed refractory multiple myeloma (RRMM) had progressed on a median of four prior lines of therapy. The overall response rate (ORR) was 0%, 43% (13/30) progressive disease, and 57% (17/30) stable disease, with a median control duration (MCD) of 3.7 months. Pembrolizumab was well-tolerated with no Grade 3 or 4 adverse events (AE) noted [26]. Contrary to advanced stage investigations, an early single-center phase I pilot trial evaluated the safety and efficacy of upfront pembrolizumab monotherapy in 13 smoldering multiple myeloma (SMM) patients with intermediate or high-risk disease. Patients received a median of eight cycles. ORR with a stringent complete response (sCR) was 8% (1/13). Otherwise, stable disease was 85% (10/13) and progressive disease was 8% (1/13). Forty-one percent (5/13) experienced immune-mediated adverse events (irAEs) of Grade 2 (G2) and G3 adverse toxicity, leading to discontinuation of therapy [25].

Several studies have evaluated whether pembrolizumab combined with immunomodulating agents (IMiDs) could enhance triplet regimen responses. Keynote-023, a phase I study, investigated the tolerability of pembrolizumab plus lenalidomide and dexamethasone in RRMM. The ORR was 44%, defined as sCR + very good partial response (VGPR) + partial response (PR). This was offset by significant toxicity, including 59.7% grade 3 or higher AEs and 3.2% deaths [27]. A single-center phase II study of 48 RRMM patients looked at response rate and toxicity of pembrolizumab plus pomalidomide and dexamethasone. ORR was 60% (29/48), CR 8% (4/48), VGPR 19% (9/48), and PR 33% (16/48). The median duration of response was 14.7 months, progression-free survival (PFS) was 17.4 months, and OS was not reached. Forty percent (19/48) of participants developed G3 or greater AEs. However, most irAEs were G2 events or lower [28]. This was followed by the phase III Keynote-183 and Keynote-185 studies, evaluating pembrolizumab plus pomalidomide and dexamethasone. RRMM with high-risk cytogenetics were randomized to pembrolizumab plus standard of care (SOC) of pomalidomide and dexamethasone vs. SOC in Keynote-183. In total, 125 were randomized to the treatment arm versus 124 in the control arm. Median follow-up was 8.1 months with a median PFS of 5.6 months. HR, 1.53 (95% CI, 1.05–2.22); *p* = 0.98. Median TTP of 8.1 vs. 8.7 months. Median overall survival (mOS) was not reached vs. 15.2 months; HR, 1.61 (95% CI, 0.91–2.85); *p* = 0.95. AEs G3/4 were 63%, including 3% (4/125) attributed to treatment deaths from irAEs, including one myocarditis and one Stevens–Johnson syndrome [29]. Keynote-185 investigated pembrolizumab in the frontline setting of newly diagnosed high-risk transplant-ineligible MM. Of 301 patients, 151 received pembrolizumab, pomalidomide, and dexamethasone with an ORR of 64%, compared to 62% in the control arm. Median TTP and PFS were not reached. HR 1.22 (95% CI, 0.67–2.22); *p* = 0.75. mOS; HR, 2.06 (95% CI, 0.93–4.55); *p* = 0.97 [30]. As a conclusion here, pembrolizumab as a single agent or in combination is not associated with meaningful response, yet is associated with significant side effects.

A phase II multicenter study of RRMM treated with a combination of nivolumab and daratumumab with (NDc) or without (ND) low-dose cyclophosphamide, demonstrated an ORR of 50% in both arms. A stable disease rate of 85% was seen with ND vs. 80% for NDc. Twenty-five percent (10/40) died from progressive disease. Median follow-up of surviving patients was 8.6 months (range 5.0–16.1). Higher infection rates were noted in the cyclophosphamide arm, 50% (20/40). Two patients died during NDc treatment: 2.5% (1/40) from cardiac arrest and 2.5% (1/40) from an *Aspergillus fumigatus* infection [31]. Several other studies showing similar outcomes of nivolumab as compared to pembrolizumab.

With the advent of PD-L1-targeted immunotherapy with atezolizumab and durvalumab, additional investigations have been published. A small phase Ib trial consisting of 24 patients treated with 1–3 prior lines of therapy studied atezolizumab in combination with daratumumab with or without the addition of lenalidomide or pomalidomide in the RRMM setting. An ORR of VGPR or greater was observed in 50% (3/6) in the combination atezolizumab and daratumumab arm vs. 43% (3/7) in the atezolizumab, daratumumab, and lenalidomide arm vs. 67% (4/6) in the atezolizumab, daratumumab, and pomalidomide arm. AE G3 or G4 was noted in 33–75% in the ICI-daratumumab arm, 86% in the ICI-daratumumab-lenalidomide arm, and 100% in the ICI-daratumumab-pomalidomide arm. The sample size was too small to draw a reliable conclusion [32]. Alternatively, a combination of durvalumab with daratumumab-refractory in RRMM was investigated in an open-label phase II prospective, single-arm, multicenter study, demonstrating stable disease in 44% (8/18) with a median duration of disease stabilization of 55 days ranging from 21–105 days. Fifty percent (9/18) developed PD immediately after the first cycle. Median duration of follow-up in surviving patients was 88 days and ranged from 14–175. The median PFS was 30 days (95% CI, 28–32) [33]. The point taken from PD-L1 trials, though incorporating ant-CD38 monoclonal antibody, was that clinical outcomes remain limited as compared to PD-1,; however, caution should be exercised in cross-trial comparisons. A summary of clinical trials is illustrated in Table 1.

Other ongoing studies include CA209-755, a phase I/II safety study evaluating nivolumab as monotherapy or in combination regimens across various relapsed and refractory hematologic malignancies. Two cohorts of this study are evaluating nivolumab in combination with daratumumab or daratumumab plus pomalidomide and dexamethasone, in patients with RRMM. The trial enrolled approximately 375 patients from 31 sites in 6 countries, including the United States [NCT03184194]. CA204142 is a phase II, multiple cohort study, assessing the safety and efficacy of elotuzumab in combination with pomalidomide and low-dose dexamethasone (EPd) vs. elotuzumab in combination with nivolumab in the RRMM setting or refractory to prior treatment with lenalidomide [NCT02612779]. CheckMate-602 is an open-label, randomized phase III trial evaluating combinations of nivolumab, elotuzumab, pomalidomide, and dexamethasone in the RRMM setting [34]. In the frontline setting of newly diagnosed MM, a phase I/II study investigated durvalumab in combination with lenalidomide with or without dexamethasone [NCT02685826] [35].

## 6. Future Aspects of Checkpoints Inhibitors

ICIs in MM have been underwhelming, with several clinical trials being suspended by pharmaceutical sponsors or the FDA; on the other hand, many remain active and are recruiting patients to understand not only the safest and most effective combination with other myeloma treatments, but also the timing of ICIs, upfront, post auto-HSCT, maintenance, or relapse refractory.

Paiva et al. demonstrated that patients with persistent MRD disease (not MRD negative) have higher PD-L1 expression [23], raising a question of using PD-L1 as single agent or in combination with non-IMiDs as a maintenance approach in MRD-persistent disease. We have a clear toxicity evidence for combining ICI with IMiDs. As some experts recommend a proteasome inhibitor as maintenance for a high-risk disease, combining proteasome inhibitors and ICI might be promising but requires further investigation.

Exploring different biological combination will be the next step to finding a place for ICI in MM treatment landscape. Venetoclax, a B-cell lymphoma 2 (BCL-2) inhibitor, has shown anti-myeloma activity—especially in patients with *t*(11; 14)—however, it comes with more infectious complications leading to higher mortality in some trials [36,37]. This reinforces the concept of individualizing treatment to maximize clinical response in order to justify risk of adverse events. A study by Kohlhapp et al. demonstrated an important potential combination for venetoclax and ICI; they showed a synergistic effect of this combination by increasing PD-1 expression in effector T-memory cells, which will open the way to moving this combination to early phase clinical trials [38].

Patients who progress on anti-CD38 monoclonal antibodies have limited treatment options and are encouraged to enroll in clinical trials, with no evidence that ICI might desensitize daratumumab-resistant MM [33], indicating that we need to avoid the duplication of trials with different ICI and search for another novel approach.

Another innovative approach has been to study the combination of PD-1 inhibitors and radiation therapy. Emory University is conducting a clinical trial examining the safety of pembrolizumab concurrently with low-dose radiation in relapsed refractory disease and accessing response/recurrence by PET-CT scans at 6 and 12 months [NCT03267888].

In order to find the best place for ICI in MM, we need to better understand the failure mechanisms. MM is a heterogonous disease and efficacy of ICI depends on several factors other than the tumor cells, such as the presence of activated effector T-cells, tumor mutational burden, enhanced antigen-presenting tumor cells, and others [39].

## 7. Conclusions

Whilst the data for ICIs for myeloma thus far have been underwhelming, the importance of immune regulation in the development and progression of MM cannot be understated, thus, providing a biological rationale for ongoing research. ICIs are unlikely to adequately treat high disease states; however, they may form an important component of therapy designed to prevent progression or relapse post-treatment, such as in MRD-positive patients, and enhance the efficacy of other immunotherapeutic agents. This may come at the cost of significant irAEs, as seen in clinical trials. Further data are required to identify mechanisms to help mitigate these risks. The results from ongoing clinical trials evaluating ICIs in myeloma are eagerly awaited. These data will help determine if ICIs become a permanent fixture in the armamentarium against myeloma.

## Figures and Tables

**Table 1:** Clinical Trial Results with PD-1/PD-L1 in Multiple Myeloma.

Trial	Number of	Phase	Treatment	Median Prior Line of Treatment	ORR %	CR %	PR %	SD %	Median Survival Results (Months) SD/PFS	Median	IrAE
	Patients									OS	
Keynote 013	30	1b RRMM	Single Arm: Pembro every 3 weeks	4	0	NA	NA	17	3.7 SD	20.2 months (14.1–NR)	40% (all grades) Gr 3 Myalgia 3.3%
Ribrag et al.											
Keynote 023	62	1 RRMM	Single Arm: Pembro + Len + Dex	4	22	2	20	25	7.2	NR	12.9% Gr 2
Mateos et al.											
Keynote 185 Zafar Usmani et al.	151 vs. 150	III—Trial Halted by FDA	Arm A: Pembro + Len + Dex	3	64 vs. 62	3.3 vs. 3.3	60 vs. 57	20 vs. 23	82% vs. 85% at 6-month HR 1.22 p 0.75	87.2% vs. 93.9% at 6-month HR 2.06 p 0.97	8% Fever and Nephritis
			Arm B: Len + Dex								
Badros et al.	48	II RRMM	Single Arm: Pembro + Pom + Dex	3	60	8	52	29	17.4 95% CI 9.2–17.5	NR	6% Pneumonitis
									p 0.04		
Mansanch, E.E. et al.	13	1 Smoldering MM	Single Arm: Pembro + Pom + Dex	NA		8 (1 patient)	NA	85	NA	NA	16% Hepatitis and Nephritis
Keynote 183 Mateos et al.	125 vs. 124	III—trial halted by FDA 2017	Arm A: Pembro + Pom + Dex.	3	34.4 vs. 40.3	0.8 vs. 0	33.2 vs. 40.3	30.4 vs. 28.2	5.6 vs. 8.4 HR of 1.53	NR vs. 15.2 HR of 1.61	4% Pneumonitis
			Arm B: Pom + Dex						(p 0.98)	(p 0.95)	
D’Souza et al.	29	II Post Auto Transplant	Single arm post-transplant day 14 +/− 4	1	31	31	NA	NA	83% at 2 years	NR	66-all grades
			EOT–CR Rate > 50%–with 9 doses								17-Gr 3
Thanendrarajan et al.	13	Retrospective RRMM	4/12 single agent	6	0 in Nivo alone	NA	NA	NA	5 weeks	5 months	IrAE was not specifically reported
			8/12 in combination		17% in combination						
Verkleji et al.	Arm A = 20	II—RRMM	Arm A: Nivo + Dara	3	Arm A = 50%	Arm A: None	A = 50	A = 15	NR	NR	No reported IrAE
	Arm B = 20		Arm B Nivo + Dara + Cyclophosphamide		Arm B = 50%	Arm B = 10	B = 40	B = 10			
Cho, H.J. et al.	Atezo Dara Combination in 6 arms Please refer to Results section text for details										
Lonial et al.	Durva + len + Dex combination: Enrollment discontinued by FDA on September 2017 no data available										
CheckMate 602	Nivolumab + Elotuzumab + Pom + Dex: Enrollment discontinued by FDA on September 2017 no data available										

Abbreviations: RRMM: Relapsed/Refractory Multiple Myeloma, Pembro: Pembrolizumab, Len: Lenalidomide, Dex: Dexamethasone, Dara: Daratumumab, Nivo: Nivolumab, Pom: Pomalidomide, Atezo: Atezolizumab, Durva: Durvalumab, EOT: End of Treatment, IrAE: immune related adverse events, CTCAE v5.0 immunotherapy adverse event Gr: grade, NA: not applicable, NR: not reached.
